# So Many Choices, How Do I Choose? Considerations for Selecting Digital Health Interventions to Support Immunization Confidence and Demand

**DOI:** 10.2196/47713

**Published:** 2023-05-24

**Authors:** Sarah Cunard Chaney, Patricia Mechael

**Affiliations:** 1 HealthEnabled Washington, DC United States

**Keywords:** immunization, social listening, mobile messaging, service delivery, low- and middle-income countries, LMIC, vaccines, demand, mHealth, vaccine confidence, public health system, vaccination, children, community health, digital health intervention, health promotion

## Abstract

Childhood vaccines are a safe, effective, and essential component of any comprehensive public health system. Successful and complete child immunization requires sensitivity and responsiveness to community needs and concerns while reducing barriers to access and providing respectful quality services. Community demand for immunization is influenced by multiple complex factors, involving attitudes, trust, and the dynamic relationship between caregivers and health workers. Digital health interventions have the potential to help reduce barriers and enhance opportunities for immunization access, uptake, and demand in low- and middle-income countries. But with limited evidence and many interventions to choose from, how do decision makers identify promising and appropriate tools? Early evidence and experiences with digital health interventions for immunization demand are presented in this viewpoint to help stakeholders make decisions, guide investment, coordinate efforts, as well as design and implement digital health interventions to support vaccine confidence and demand.

## Introduction

Many communities around the world continue to face barriers to accessing routine childhood immunization services, resulting in an estimated 18 million children worldwide who do not receive any lifesaving vaccines (zero-dose children) and an additional 25 million who do not complete the recommended vaccination schedule [1]. Challenges and barriers may include access to quality and reliable services, financial burdens, or a reluctance to seek vaccination because of distrust, negative experiences, or concerns over safety and side effects [2-4].

The majority of unvaccinated and undervaccinated children live in low- and middle-income countries, highlighting the pressing need to prioritize sustainable improvements in immunization programs and approaches to increase community acceptance and access to lifesaving vaccines in the poorest and most vulnerable countries in the world [1]. In addition to safe and effective vaccines and an efficient supply chain, a successful immunization program also requires communities and individuals who are willing and able to receive vaccines for themselves and their children [5]. The COVID-19 pandemic focused attention on the importance of vaccine confidence and demand for immunization, whereby many countries witnessed a growing distrust toward recommended prevention measures and the government, health systems, and institutions delivering new vaccines [6,7].

Immunization demand refers to individuals and communities with a positive attitude toward vaccines, positive perceptions about the quality of services available to them, and actively seek out and advocate for others to use these services [8]. Rumors and distrust may flare up at any time, not only during a pandemic or novel health emergency. This reflects the complex process involved in vaccine decision-making, influenced by personal experience, perceptions of the institutions involved, and confidence in those institutions [9,10]. The local community, health system, media environment, social norms, and individual perceptions operate in an iterative cycle to influence vaccine uptake and demand for immunization.

## Digital Health Interventions for Immunization Confidence and Demand

Digital health interventions are increasingly being used to promote immunization uptake as mobile devices and computer technology are more common in everyday life [11,12]. Reviews of early experiences and evidence demonstrate the potential of digital technologies, mobile messaging services, and data generated from immunization program activities to promote community demand for immunization [13-15]. However, due to inconsistent results and variation in local contexts, there is no single solution or straightforward blueprint for applying digital solutions to vaccine confidence and demand.

Gavi, the Vaccine Alliance provides lifesaving vaccines to more than 800 million children in over 70 countries. Their recent Digital Health Information Strategy recognizes “digital interventions supporting vaccine confidence and demand for immunization” as one of the 6 most promising intervention areas to achieve improved immunization outcomes [16]. In collaboration with UNICEF (United Nations International Children’s Emergency Fund), the World Health Organization, the Vaccination Demand Hub, and HealthEnabled, a recent series of reviews provide recommendations for country immunization programs to identify appropriate digital health interventions that can help promote vaccine confidence and immunization demand. These efforts inform 2 complementary resources to guide the use of digital health interventions for vaccine confidence and immunization demand.

The first resource, “Finding the signal through the noise: a landscape review and framework to enhance the effective use of digital social listening for immunisation demand generation” [17], presents a systematic process for applying digital social listening to increase and sustain vaccine confidence. The second resource, “Digital health information interventions for immunisation demand generation: a guide for selecting appropriate tools and technologies” [18], presents a stepwise approach and important considerations for decision makers and stakeholders in low- and middle-income countries. These resources, along with the commitment of international donors, early evidence, and documented experiences, point to the potential for digital health interventions to boost immunization demand as part of a comprehensive immunization program. However, the success of any program will depend on careful planning, formative research, and meaningful user engagement.

Key intervention areas are highlighted below to help designers, implementers, and policy makers understand the range of use cases and considerations for effective planning, selection, and design of digital health interventions for immunization demand generation.

## Journey to Health and Immunization

**Figure 1:**
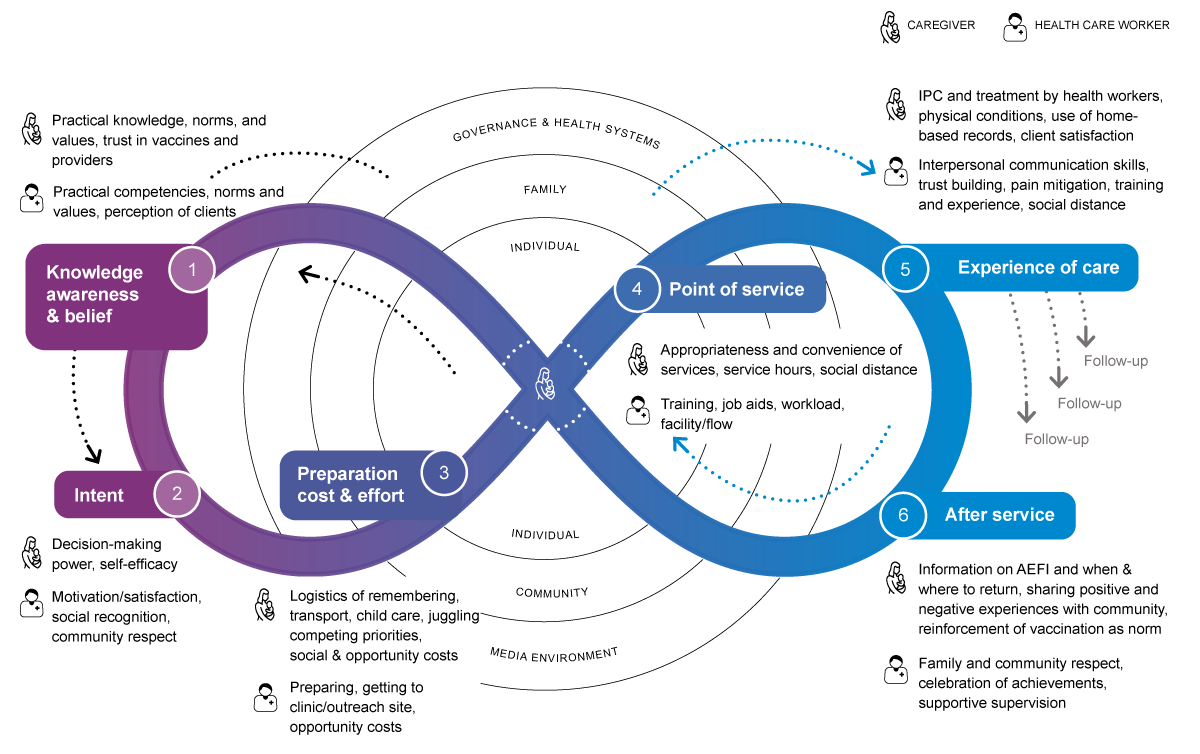
The Journey to Health and Immunization provides a systematic approach to help understand bottlenecks and identify possible interventions that can address existing problems in a particular community, infrastructure, or immunization system (originally published and adapted from UNICEF’s Human Centered Design 4 Health [19]). AEFI: adverse event following immunization; IPC: interpersonal communication.

UNICEF’s Journey to Health and Immunization (Figure 1) is a useful framework to help understand different factors that influence the vaccine decision-making process [19]. It is intended to help program planners and implementers identify unique situations and help overcome barriers faced by both caregivers and health workers in the target community, society, and local context.

The Journey to Health and Immunization framework represents 2 connected journeys—one from the perspective of the caregiver and the other from the perspective of the health worker. These 2 profiles are key to strong immunization demand, but each one experiences different pressures, opportunities, and challenges along their journey.

The caregiver’s Journey is influenced by behaviors, costs, and feelings associated with the decision to seek out vaccination services. These factors are influenced by the surrounding cultural norms, family practices, and past experiences with the health system.

The health worker’s journey includes the work environment, training, and interactions with the community that contribute to their job satisfaction and motivation. A health worker’s enthusiasm, skills, and experience will impact their interactions with caregivers and, in turn, the community’s demand for immunization services. Health workers are the face of the immunization program and are critical to supporting strong immunization demand.

A useful resource for helping to understand the local context and barriers to uptake in a community are the Behavioral and Social Drivers of Vaccine Uptake Tools [20]. These standardized questions examine the situation at the facility and household levels to help programs measure underlying barriers, inform program planning, and target demand and communication interventions [21].

Any solution or approach to increase demand will depend on current challenges and needs of the community. In some cases, a digital health intervention may be able to help address prioritized immunization demand needs. Digital technologies and tools provide opportunities to understand community perceptions and concerns, extend the reach of immunization messages, and provide support for health workers to do their job.

## Digital Social Listening to Understand Vaccine Sentiment

Social listening is the systematic monitoring of public discourse and sentiment as expressed in digital media, computer- and web-based sources of information, as well as print and traditional media, representing different populations and geographies [22]. If identified barriers to vaccine demand in the community involve a lack of public trust, dissatisfaction with services, fears, or misinformation, social listening may be a useful tool to consider. Public web-based social media can be monitored to track conversations in near real time with new digital media monitoring tools that can help bring potentially harmful rumors to the attention of the government immunization program [23]. Public feedback mechanisms and digital social listening can provide actionable insights that can then be used to inform community engagement strategies, policies, communication campaigns, and quality improvement activities. The changes enacted in response to social listening may apply to any of the 6 steps along the Journey to Health and Immunization to improve community access and demand for immunization services.

In recent experiences with COVID-19, the need to manage the spread of harmful misinformation contributed to targeted information campaigns across a variety of communication channels to counteract myths and misconceptions about the virus and vaccines [24,25]. Active information gathering and feedback mechanisms use data from remote crowdsourcing apps, automated mobile phone surveys, or toll-free telephone lines and hotlines [26-29]. With dedicated resources, staff, and time allocated, some programs developed systems for gathering information about public sentiment from community volunteers, radio programs, hotlines, and other communication channels that enrich insights from digital sources [24,25].

## Digital Communication to Promote Immunization and Provide Basic Information for How to Access Immunization Services

Digital health interventions can expand the reach of the immunization system by sending reminders and reinforcing counseling messages beyond the one-on-one conversations that take place during an immunization appointment. These may take the form of simple reminders or more in-depth behavior change messages delivered by text-based SMS or prerecorded audio messages sent to the caregiver’s mobile phone. Digital communication messages, reminders, and social media engagement can respond to insights and barriers uncovered through a community assessment, social listening, or other community feedback mechanism. These approaches can help build trust and reinforce immunization as a positive social norm (step 1), counter misinformation (step 2), provide nudges (step 3), and practical information on where and when to access services (step 2). Digital technologies and tools can be useful components of an immunization communication and outreach strategy when supported with formative research into demand-side challenges for vaccine uptake.

Evidence and experiences from various settings show that mobile phone reminders and recall messages can have a positive impact on immunization uptake, vaccination coverage, and timeliness [30-34]. Social and behavior change communication programs have demonstrated improvements in maternal and child health service uptake and knowledge, including knowledge about routine childhood immunization, increased intent to vaccinate children, and in some studies, a significant increase in complete vaccination [26,35-39].

Digital technologies also have the power to amplify creative and persuasive storytelling that can establish immunization messages as a social norm. Immunization programs can use storytelling through social media or microinfluencers to change intention, attitudes, and behaviors [40]. Many immunization programs have documented experiences and strategies with social media engagement as a response to the COVID-19 infodemic reaching large populations with targeted solutions and communication approaches [9,41,42]. Audio messages can also be used to reach communities with lower literacy rates in local languages and have the advantage of presenting well-known local voices to bring credibility to the messages and engage the audience.

## Digital Support Tools for Health Workers to Provide Quality Immunization Services

A health worker’s motivation and job performance are important parts of maintaining demand for immunization services in the community. The work environment, training opportunities, as well as respect from clients, coworkers, and supervisors contribute to job satisfaction and motivation. eLearning, digitally delivered health worker training, remote supervision, and digital job aids have the potential to improve the quality of immunization services, boost health workers’ skills and motivation, and improve the overall immunization experience for caregivers. Digital applications for health works can help support their knowledge, awareness, and beliefs (step 1); confidence, motivation, and satisfaction (step 2); provide quality services (steps 4 and 5); and provide supportive supervision (step 6). These are all key considerations that influence overall vaccine confidence and demand in the community and are crucial to developing a health workforce that reinforces positive experiences in the community.

Digital health interventions can facilitate remote supervision and peer-support and provide skills and refresher training with technical content about new vaccines or health emergencies. They can be especially useful for remote locations where opportunities to travel for in-person training are limited. Curriculum and training modules for health workers have been adapted for mobile phones, tablets, or other available digital devices [43-45]. Decision-support tools and reminders for health workers in maternal and child health programs have demonstrated improvements in quality of care, client satisfaction, adherence to protocols, health worker workflow management, as well as confidence and respect in the community [46-51]. Mobile phone–delivered and electronically delivered decision support and job aids for health workers can help them correctly schedule routine and catch-up immunization appointments and reduce missed opportunities for vaccination [52,53].

## Challenges and Limitations

Despite many digital technologies and approaches that show potential to address vaccine confidence and demand barriers, no single intervention can reach every person or solve all the complex challenges involved in vaccine acceptance and uptake. Digital immunization demand solutions may not be feasible or appropriate in many situations.

Mobile phone ownership and culture of use within the existing digital infrastructure as well the cost and coverage of mobile network operators will play a critical role in any digital health intervention [54]. Literacy levels, the gender digital divide, and gender norms will influence access to technology, agency, and use of data among both caregivers and health care workers [55]. The same digital and mobile communication channels used to reach beneficiaries with accurate and relevant information about vaccines can also be used to access misinformation, spread rumors, and create confusing messages that seed doubt and mistrust. These and other challenges and limitations must be considered through careful planning and assessment of the local context and needs of the community. No tool, recommendation, or lesson will fit every possible scenario.

Digital health interventions to support immunization demand are just one part of a comprehensive immunization program strategy and quality improvement approach. Creating genuine demand for immunization services will not come from simple one-way education and communication campaigns but requires a genuine commitment to meaningful community engagement, end-user cocreation and a willingness to make changes based on feedback, community concerns, and criticism. Digital technologies can help open channels of communication, promote transparency, and increase access to information but are only one possible tool to help improve access to quality immunization services.

## Conclusions

Digital tools and technologies have the potential to bridge the gap between what communities need and the practices of the immunization program. When digital health applications are designed in response to context-specific factors limiting vaccine uptake, they have the potential to make new connections, provide engaging content, and support quality service delivery.

This overview of digital applications for immunization confidence and demand should serve as a starting point to guide future design, implementation, and evaluation. It is also meant to draw attention to this topic and encourage program planners to strengthen the evidence base; share experiences with the broader immunization community; and document lessons, challenges, and recommendations to inform future activities.

There are considerable gaps in the literature, and more robust evidence and documentation of experiences are necessary to support widespread application of digital technologies for immunization demand in low- and middle-income countries. Qualitative and quantitative research should use standard definitions, indicators, and methodologies to support a better understanding of effective digital approaches over time and across different contexts. The Principles for Digital Development are recommended to ensure meaningful user-centered engagement [56]. Any introduction of new digital tools should include parallel assessment and investment to strengthen the digital ecosystem, governance, capacity, and leadership in the country to help ensure a strong foundation for future applications [16,57].

Dedicated resources and a long-term commitment are essential for effective digital immunization demand programing. Gavi’s guide for selecting appropriate tools and technologies for immunization demand generation [18] can help country programs as well as developers and policy makers to select appropriate digital solutions to address vaccine confidence and demand with useful suggestions and lessons from early experiences.

With appropriate planning and investment, technologies for digital immunization demand can become useful mechanisms to promote immunization confidence and demand and help achieve the goal of providing the full schedule of lifesaving vaccines to all children everywhere.

## Abbreviations

GAVI: Gavi, the Vaccine Alliance
UNICEF: United Nations Children’s Emergency Fund
